# Self-Powered Pressure–Temperature Bimodal Sensing Based on the Piezo-Pyroelectric Effect for Robotic Perception

**DOI:** 10.3390/s24092773

**Published:** 2024-04-26

**Authors:** Xiang Yu, Yun Ji, Xinyi Shen, Xiaoyun Le

**Affiliations:** 1 School of Physics, Beihang University, Beijing 100191, China; 2 Beijing Advanced Innovation Center for Big Data-Based Precision Medicine, School of Medicine and Engineering, Beihang University, Beijing 100191, China; 3 Beijing Key Laboratory of Advanced Nuclear Energy Materials and Physics, Beihang University, Beijing 100191, China; 4 Department of Electrical and Computer Engineering, National University of Singapore, 4 Engineering Drive 3, Singapore 117583, Singapore

**Keywords:** piezo-pyroelectric effect, PMN-PT, self-powered sensor, temperature sensing, pressure sensing

## Abstract

Multifunctional sensors have played a crucial role in constructing high-integration electronic networks. Most of the current multifunctional sensors rely on multiple materials to simultaneously detect different physical stimuli. Here, we demonstrate the large piezo-pyroelectric effect in ferroelectric Pb(Mg_1/3_Nb_2/3_)O_3_-PbTiO_3_ (PMN-PT) single crystals for simultaneous pressure and temperature sensing. The outstanding piezoelectric and pyroelectric properties of PMN-PT result in rapid response speed and high sensitivity, with values of 46 ms and 28.4 nA kPa^−1^ for pressure sensing, and 1.98 s and 94.66 nC °C^−1^ for temperature detection, respectively. By leveraging the distinct differences in the response speed of piezoelectric and pyroelectric responses, the piezo-pyroelectric effect of PMN-PT can effectively detect pressure and temperature from mixed-force thermal stimuli, which enables a robotic hand for stimuli classification. With appealing multifunctionality, fast speed, high sensitivity, and compact structure, the proposed self-powered bimodal sensor therefore holds significant potential for high-performance artificial perception.

## 1. Introduction

Robotic perception facilitated by a variety of sensors enables robots to interact with their surroundings safely and accurately, playing a vital role in the field of robotic manipulation. Various sensors, particularly strain and force sensors based on hierarchical and gradient piezoelectric composites, have attracted considerable attention due to their ability to detect mechanical stimuli in the environment [1,2,3,4,5]. For instance, materials such as hierarchical porous poly(vinylidene fluoride)/BaTiO_3_ foams [6], polyvinylidene fluoride hexafluoropropylene/ZnO composite nanaofibers [7], gradient carbon nanotube/polyvinylidene fluoride composites [8], and hierarchical PbZr_1–x_Ti_x_O_3_/poly(vinylidene fluoride-trifluoroethylene) ceramic textiles show great promise for monitoring mechanical stimuli [9,10,11,12]. However, sensors that solely monitor mechanical stimuli are inadequate for providing a comprehensive perception of the complex environment, which simultaneously involves multiple physical stimuli [13,14,15]. Multifunctional sensors with compact configurations and low consumption play a significant role in solving this problem. Developing multifunctional sensors for simultaneously monitoring multiple physical parameters, such as temperature, pressure, speed, light intensity and humidity, has become an urgent topic to be investigated. So far, a variety of materials have been explored for realizing multifunctional sensing, including spin-rich van der Waals semiconductor films [16], organic thermoelectric composite materials [17], organic–inorganic hybrid sponges [18,19], thermogalvanic hydrogels [20], and ferroelectric materials [21,22].

Among these materials, ferroelectric materials with large dielectric permittivity and high spontaneous polarization exhibit piezoelectric, pyroelectric, triboelectric, flexoelectric and photovoltaic properties, which makes them capable of detecting different kinds of physical stimuli without any external energy suppliers [23,24,25,26]. Moreover, ferroelectric materials are viewed as one of the most promising candidates for constructing high-integration multifunctional sensors owing to the coexistence of piezoelectric, pyroelectric, photovoltaic, flexoelectric and triboelectric properties [27,28,29,30,31]. Ferroelectric Pb(Mg_1/3_Nb_2/3_)O_3_-PbTiO_3_ (PMN-PT) single-crystal material is a solid solution formed between Pb(Mg_1/3_Nb_2/3_)O_3_ and PbTiO_3_ materials. By controlling the molar ratio of Pb(Mg_1/3_Nb_2/3_)O_3_ and PbTiO_3_, PMN-PT single crystals with large piezoelectric coefficient, high dielectric permittivity and strong electromechanical coupling effect can be achieved [32,33,34]. Ferroelectric PMN-PT single-crystal materials have been intensively investigated for constructing piezoelectric devices owing to their excellent piezoelectric property [35,36,37]. In addition, ferroelectric PMN-PT single-crystal materials are expected to have a high pyroelectric coefficient at room temperature, showing great potential for monitoring thermal fluctuations [38,39,40]. With both competitive piezoelectric and pyroelectric characteristics, PMN-PT single crystals are promising for simultaneously detecting force and thermal stimuli. However, multifunctional self-powered sensors based on PMN-PT single crystals are still remaining to be developed.

In this work, we present a pressure–temperature bimodal self-powered sensor based on the large piezo-pyroelectric effect of ferroelectric PMN-PT single-crystal materials. With a simple three-layer structure, the bimodal sensor can be utilized for rapid pressure and temperature sensing with high sensitivities of 28.4 nA kPa^−1^ and 94.66 nC °C^−1^, respectively. In addition, by leveraging the distinct differences in the response speed of piezoelectric and pyroelectric effects, the piezo-pyroelectric effect of PMN-PT can be utilized to simultaneously detect the pressure and temperature variation from mixed-force thermal stimuli. Based on the measured pressure and temperature information by the bimodal PMN-PT sensor, a high-accuracy robotic stimuli classification task can be achieved. The excellent multifunctional sensing performance, together with the simple configuration makes the self-powered bimodal PMN-PT sensor hold great potential for highly precise and low-consumption artificial perception applications.

## 2. Materials and Methods

### 2.1. Preparation of the Bimodal PMN-PT Sensor

The commercial (001)-oriented PMN-PT single crystal with a PMN:PT molar ratio of 7:3 (Hefei Kejing Materials Technology Co., Ltd., Hefei, China) was utilized as the functional component of the bimodal sensor. The Ag electrodes were deposited on both sides of the PMN-PT using the DC magnetron sputtering technique at room temperature in an Ar atmosphere. The sputtering power density, pressure, and time were set to 1.52 W cm^−2^, 0.35 Pa and 1800 s, respectively, resulting in an approximate thickness of 1 μm for the Ag electrodes. After being polarized in silicon oil at 2.5 kV for 30 min at room temperature, the Ag/PMN-PT/Ag device was directly utilized for performance evaluation. The PMN-PT sensor for demonstrating robotic hand perception is covered with a thin polyimide film using silver paste and integrated into an acrylic frame.

### 2.2. Characterizations of the Bimodal PMN-PT Sensor

The dielectric property was measured by a Partul DMS500 dielectric temperature spectrometer (Partulab Technology Co. Ltd., Wuhan, China). The piezoelectric constant was tested by a ZJ-4AN quasi-static piezoelectric coefficient testing meter (Institute of Acoustics, Chinese Academy of Sciences, Beijing, China). The polarization–electric field (*P*-*E*) hysteresis loops were measured utilizing a Sawyer–Tower circuit at a frequency of 5 Hz. The press stimuli were applied and monitored by a homemade system which is composed of a MX2-500N single-column dynamometer (Imada Co., Ltd., Toyohashi, Japan) and a ZTA-50N dynamometer (Imada Co., Ltd., Toyohashi, Japan). The temperature was monitored by an Optris PI400 IR thermographic camera (Optris GmbH, Berlin, Germany). The charge and current were recorded by a Keithley 6514 Electrometer and a Keithley 2611B system source meter, respectively.

## 3. Results

### 3.1. Design Concept and Device Structure

**Figure 1:**
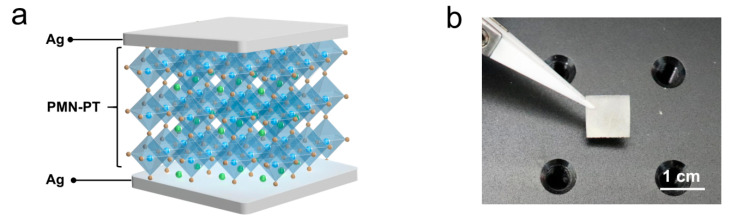

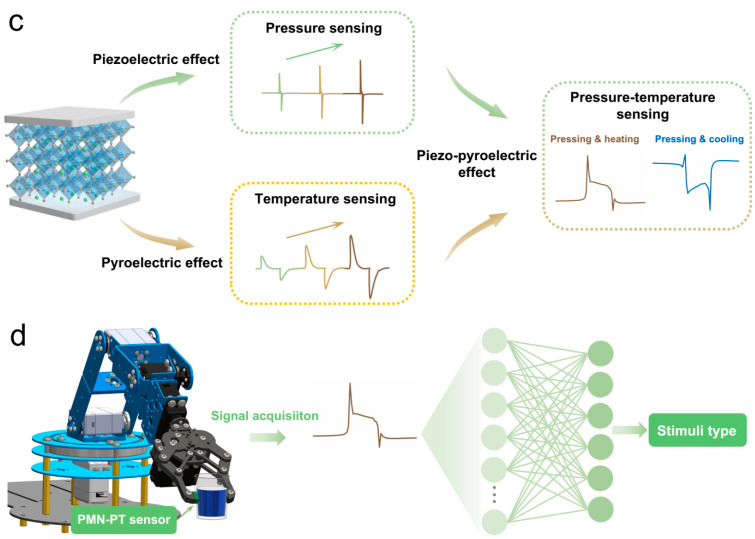
Design and functions of the self-powered bimodal sensor. (**a**) Schematic diagram of the bimodal sensor with Ag/PMN-PT/Ag structure. (**b**) A photographic image of the bimodal sensor. (**c**) Illustrations of the bimodal sensor for individual/simultaneous pressure and temperature sensing. (**d**) Schematic of a robotic stimuli classification system based on the bimodal sensor.

Figure 1a illustrates the schematic diagram of the pressure–temperature bimodal self-powered sensor, which is composed of a ferroelectric PMN-PT thin disk sandwiched between Ag top and bottom electrodes. The PMN-PT sensor has a square shape with a side length of 10 mm and a thickness of 500 μm, as shown in the photographic image in Figure 1b. Figure S1a exhibits the room-temperature *P*-*E* hysteresis loops of the PMN-PT sensor, and Figure S1b shows the dependence of the remnant polarization on the strength of the electric field. It is obvious that as the strength of the applied electric field increases, the *P*-*E* loops gradually saturate, and the remnant polarization increases, indicating the good ferroelectric properties of the PMN-PT materials. Figure S2 presents the dielectric properties of unpolarized and polarized PMN-PT materials at different frequencies as a function of temperature. The dielectric permittivity of the unpolarized PMN-PT increases with the temperature (Figure S2a), exhibiting an evident peak near 135 °C denoting the Curie transition temperature (ferroelectric phase to paraelectric phase) [41]. However, the dielectric permittivity of the polarized PMN-PT exhibits a shoulder peak near 105 °C, together with a maximum peak at around 135 °C (Figure S2b). The shoulder peak is ascribed to the transition from the macrodomain to the microdomain, and the maximum peak represents the transition from the ferroelectric phase to the paraelectric phase (Curie transition) [42,43]. The high transition temperatures of the PMN-PT enable the sensor to operate in a wide temperature range, which is sufficient for daily applications. Moreover, the PMN-PT materials exhibit low dielectric loss (Figure S2c,d), facilitating highly efficient energy conversion for sensing applications. A well-polarized PMN-PT sensor demonstrates a high piezoelectric constant *d*_33_ with an absolute value of 1369 pC N^−1^ (Figure S3), paving the way for high-performance pressure sensing. Furthermore, the PMN-PT device exhibits strong pyroelectricity with a large pyroelectric coefficient of approximately 95 nC cm^−2^ °C^−1^ (Figure S4), which enables highly sensitive temperature detection. Simultaneously possessing considerable piezoelectricity and pyroelectricity, the PMN-PT sensor is well suited for individual/simultaneous temperature and pressure monitoring based on the piezoelectric effect, pyroelectric effect and piezo-pyroelectric effect, as illustrated in Figure 1c. Equipped with the bimodal pressure–temperature sensor, a robotic hand that is able to classify stimuli types can be realized, as shown in Figure 1d. When grasping objects, the robotic hand can effectively perceive the pressure and temperature via the generated piezo-pyroelectric signals from the PMN-PT bimodal sensor. With the assistance of an artificial neural network (ANN), the stimuli type induced by the grasping can be classified by analyzing the pressure and temperature features contained in the piezo-pyroelectric signals, which paves the way for precise object manipulation and classification.

### 3.2. Self-Powered Pressure Sensing

**Figure 2:**
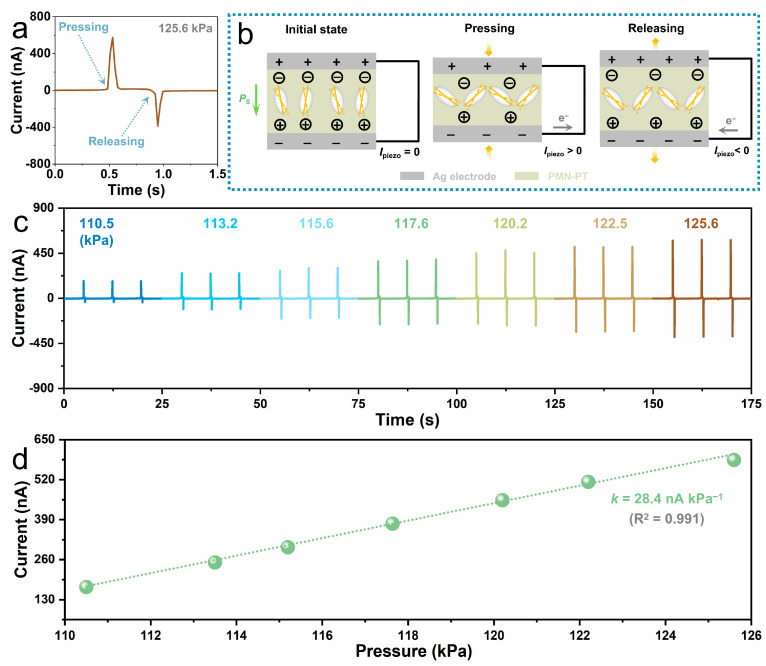
Self-powered pressure sensing performance of the PMN-PT bimodal sensor. (**a**,**b**) A typical piezoelectric current signal of the sensor (**a**) and the corresponding current generation mechanism (**b**) under pressing stimuli with a pressure of 125.6 kPa. (**c**) Time-dependent piezoelectric current of the sensor under pressing stimuli with pressure changes from 110.5 kPa to 125.6 kPa. (**d**) Dependence of the positive piezoelectric current values on the applied pressure.

We first investigated the self-powered pressure sensing performance of the PMN-PT bimodal sensor at room temperature by using a single-column dynamometer to periodically apply pressing stimuli on the device. Figure 2a exhibits a typical time-resolved piezoelectric signal from the sensor during one pressing-releasing cycle with an applied pressure of 125.6 kPa. It is obvious that a sharp positive current is generated by pressing the sensor, and a sharp negative current is produced when releasing the stimuli. Figure 2b illustrates the generation mechanism of the observed current signals. At the initial state, the electric dipoles in the PMN-PT are well-aligned and oscillate around their aligned axes within a certain degree, leading to stable spontaneous polarization. As a consequence, positive and negative charges are attracted on the opposite Ag surfaces, reaching an equilibrium state, thus no currents are generated in the external circuit. When the sensor is pressed, the electric dipoles in the PMN-PT oscillate within a larger degree, resulting in weakened spontaneous polarization. Consequently, the attracted positive and negative charges move towards the opposite electrodes, causing a positive piezoelectric current in the external circuits. On the contrary, when the pressure is removed, the oscillation degree of the electric dipoles gets smaller; hence, the spontaneous polarization becomes strengthened. As a result, more and more positive and negative charges are attracted to the opposite electrodes, forming a negative piezoelectric current in the external circuit. The sensor exhibits a fast speed for pressure sensing, with the response time (the time taken for the current increases from its minimum to maximum) and recovery time (the time required for the current increases from its minimum to maximum) of only 46 ms and 47 ms, respectively, as shown in Figure S5. Figure 2c illustrates the time-dependent piezoelectric current under pressing stimuli with different pressures, where the piezoelectric current signals measured for three cycles under each pressure exhibit similar values, indicating the good reliability of the sensor. Additionally, a strong dependence of piezoelectric current values on pressure is observed. The positive piezoelectric current values under various pressures are summarized in Figure 2d, which exhibits a linear increasing trend with the applied pressure, indicating the positive piezoelectric current value can be an excellent indicator for monitoring pressure. The sensitivity of the sensor for pressure sensing is defined as the slope of the fitting linear line, which is 28.4 nA kPa^−1^. The electrical impedance of the sensor operated under press stimuli is also assessed according to the maximum output power [44], which is about 50 MΩ, as illustrated in Figures S6 and S7). To assess the long-term performance of the PMN-PT sensor for pressure monitoring, a pressure of 125.6 kPa was cyclically applied to the sensor for 1800 s. The resulting time-dependent output current is depicted in Figure S8a. An enlarged portion of the output current (Figure S8b) clearly shows no attenuation, indicating the remarkable robustness of the device. The cycle-to-cycle variability of the output current can be obtained by analyzing the coefficient of variation (*σ*/*µ*), where *σ* and *µ* represent the standard deviation and the mean values of the peak output current, which is only 1.43% (Figure S8c), demonstrating the excellent long-term stability of the PMN-PT sensor.

### 3.3. Self-Powered Temperature Sensing

**Figure 3:**
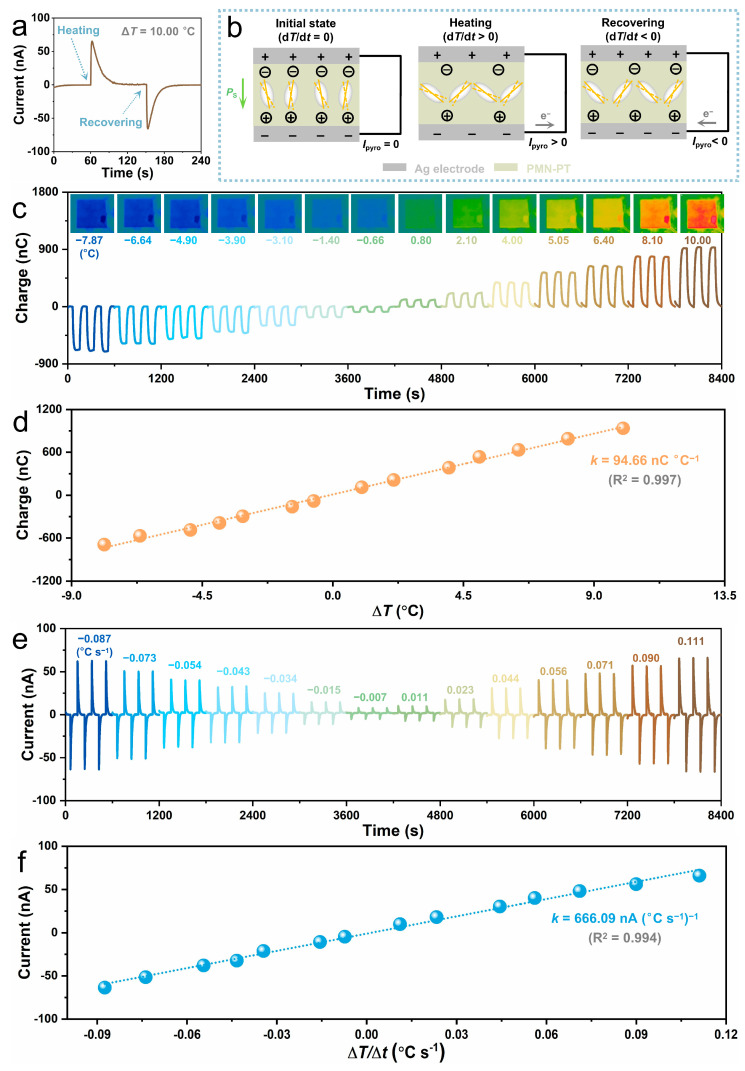
Self-powered temperature sensing performance of the PMN-PT bimodal sensor. (**a**,**b**) A typical pyroelectric current signal (**a**) and the corresponding current generation mechanism (**b**) of the sensor under heating stimuli with a temperature gradient Δ*T* of 10.00 °C. (**c**) Time-dependent output charge and infrared thermal images of the sensor under cooling/heating stimuli with the temperature gradient Δ*T* changes from −7.87 °C to 10.00 °C. (**d**) Dependence of the output current on temperature gradient Δ*T*. (**e**) Pyroelectric current of the sensor under cooling/heating stimuli with the average temperature change rate d*T*/d*t* varies from −0.087 °C s^−1^ to 0.111 °C s^−1^. (**f**) Pyroelectric current as a function of temperature change rate.

The self-powered temperature sensing performance of the PMN-PT bimodal sensor is investigated by cyclically heating and cooling the device via a semiconductor thermoelectric module. The temperature gradient Δ*T* is defined as the temperature difference of the sensor before and after cooling/heating stimuli. Figure 3a exhibits a representative time-dependent output current of the PMN-PT sensor going through a heating stimuli cycle (Δ*T* = 10.00 °C), which shows two obvious current peaks with opposite polarities. When the sensor is heated, a positive current can be detected. When the heating stimulus is removed, a negative current can be generated. The current generation mechanism of the sensor during this heating process is presented in Figure 3b. Similarly to the generation of piezoelectric signals, the generated current by heating stimuli is ascribed to the variation of spontaneous polarization. At room temperature (d*T*/d*t* = 0), the spontaneous polarization in PMN-PT remains constant, and the whole system is in an equilibrium state; hence, no current exists in the external circuit. When the sensor is heated (d*T*/d*t* > 0), oscillation of the electric dipoles in PMN-PT is intensified, leading to lower spontaneous polarization. As a consequence, the number of the attracted charges on both sides of the sensor reduces, forming a positive current in the external circuit. When the heating stimulus is removed, the temperature of the sensor gradually decreases to room temperature (d*T*/d*t* < 0), correspondingly, the oscillation of the electric dipoles is effectively suppressed. As a consequence, the average spontaneous polarization in PMN-PT is increased, generating a negative current in the external circuit. Based on this principle, the sensor shows a negative output current upon cooling (d*T*/d*t* < 0) due to enhanced spontaneous polarization, and a positive output current during its temperature recovery (d*T*/d*t* > 0) because of reduced spontaneous polarization (Figure S9). Figure 3c shows the output charges of the bimodal PMN-PT sensor under various temperature gradients Δ*T* (Figure S10). Both the polarity and values of the output charge exhibit obvious dependence on the temperature gradient Δ*T*, suggesting the output charge can be an effective indicator to reflect the variation in temperature gradient Δ*T*. In addition, the output charge corresponding to each temperature gradient Δ*T* exhibits high reproducibility, indicating the high reliability of the sensor for temperature sensing. Figure 3d illustrates the output charge of the PMN-PT sensor as a function of temperature gradient Δ*T*, which shows that the output charges linearly increase with the temperature gradient Δ*T*, indicating the good response of the sensor to temperature variation. The sensitivity for detecting temperature gradient Δ*T* is evaluated as the slope of the fitting linear line, which is 94.66 nC °C^−1^. The PMN-PT sensor shows a high-temperature resolution, even if a small temperature gradient Δ*T* of only −0.66 °C is clearly identified (Figure 3c). Further, we evaluated the performance of the PMN-PT sensor for detecting the average temperature change rate by analyzing the pyroelectric current. Figure 3e illustrates the time-dependent pyroelectric current under cooling and heating stimuli with various average temperature change rates, which shows the pyroelectric current is highly correlated to the average temperature change rate. The pyroelectric current shows linear increasing characteristics with the average temperature change rate, with a high sensitivity of 666.09 nA °C^−1^ (Figure 3f). An enlarged time-dependent pyroelectric current shows that the bimodal PMN-PT sensor has a fast temperature response speed with a response time of 1.98 s, as displayed in Figure S11. When operating for temperature sensing, the electrical impedance of the bimodal PMN-PT sensor is about 0.5 GΩ and 0.3 GΩ under cooling and heating stimuli, respectively (Figure S12). The long-term performance of the PMN-PT sensor for temperature monitoring was evaluated by periodically heating the device for more than 4 h, and the corresponding current from the sensor is demonstrated in Figure S13a. The output current signals are highly reproducible, as exhibited in Figure S13b. Statistical analysis shows that the cycle-to-cycle variation of the output current is only 1.13% (Figure S13c), indicating the excellent long-term reliability of the PMN-PT device as a temperature sensor.

### 3.4. Simultaneous Pressure and Temperature Sensing

**Figure 4:**
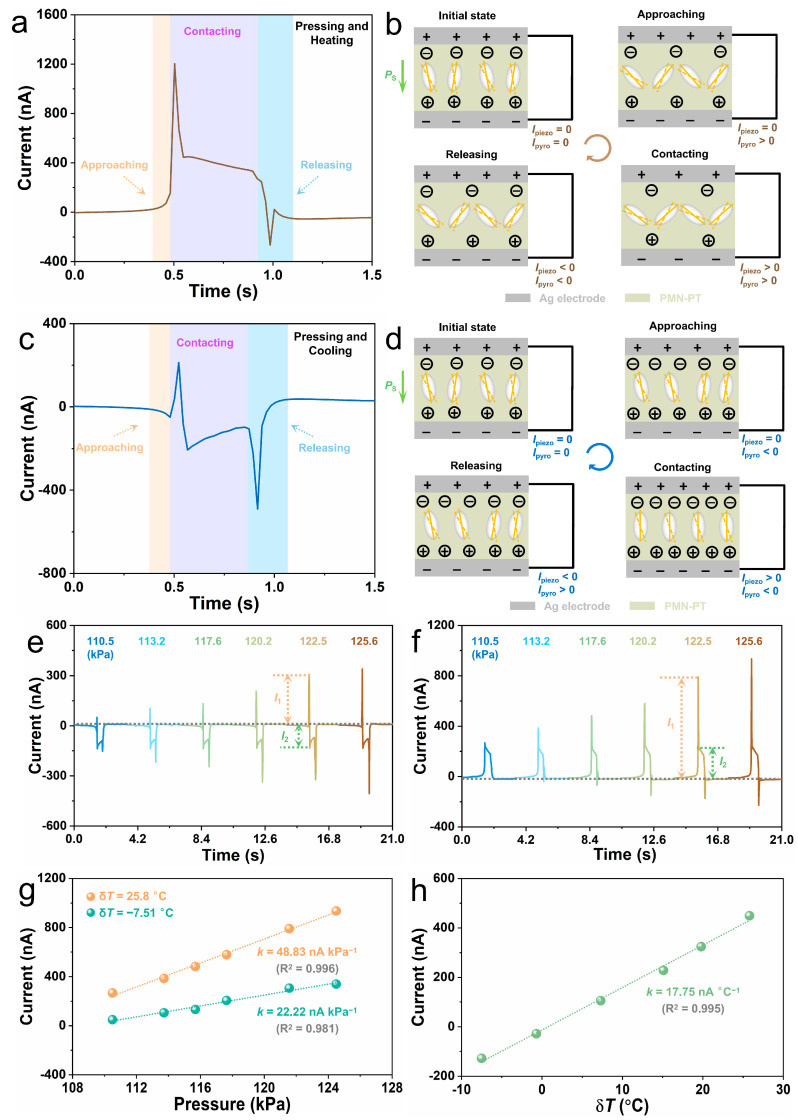
(**a**,**b**) A typical output current signal (**a**) and its generation process (**b**) as the PMN-PT sensor is pressed by a heated thermoelectric module. (**c**,**d**) A representative output current (**c**) and its generation process (**d**) as the PMN-PT sensor is stimulated by a cooled thermoelectric module. (**e**,**f**) The output current from the PMN-PT sensor under different pressures exerted by heated I and cooled (**f**) thermoelectric modules. (**g**) The peak piezo-pyroelectric current *I*_1_ as a function of pressure under various δ*T*. (**h**) The plateau piezo-pyroelectric current *I*_2_ as a function of δ*T* under various pressures.

The coexistence of large piezoelectric and pyroelectric responses in our PMN-PT bimodal sensor enables the device for simultaneous pressure and temperature sensing from mixed-force thermal stimuli. To evaluate the bimodal sensing performance of the PMN-PT sensor, a thermoelectric module with different temperatures was utilized to press the device to generate mixed force-press stimuli. Figure 4a displays a typical output current signal as the PMN-PT sensor is pressed by a heated thermoelectric module. The current signal can be divided into three stages according to the relative position between the thermoelectric module and the PMN-PT sensor, including approaching, contacting and releasing. The corresponding current generation mechanism is illustrated in Figure 4b. At the initial state, the PMN-PT sensor is in an equilibrium state with constant spontaneous polarization, and no current is generated in the external circuit. When the heated thermoelectric module approaches the PMN-PT sensor, heat is transmitted from the module to the sensor, leading to a slight increase in the temperature of the PMN-PT. As a consequence, the spontaneous polarization in the PMN-PT is reduced; thus, a small positive pyroelectric current is generated in the external circuit. As the heated thermoelectric module is in contact with the PMN-PT sensor, a strong force is immediately applied to the device, which dramatically decreases the spontaneous polarization of the PMN-PT, hence generating a positive piezoelectric current in the external circuit. Meanwhile, the close contact enables more effective heat transmission from the thermoelectric module to the PMN-PT, further decreasing the spontaneous polarization of the PMN-PT and forming a positive pyroelectric current in the external circuit. The combination of the piezoelectric and pyroelectric effects leads to a sharp positive current peak at the beginning of the contact. Due to the rapid speed of the piezoelectric response, the piezoelectric current dramatically decreases and finally vanishes, while the pyroelectric current gradually reduces. When the heated thermoelectric module is removed from the PMN-PT sensor, the force applied to the PMN-PT is released rapidly; meanwhile, the temperature of the PMN-PT starts to decrease. As a consequence, the spontaneous polarization of the PMN-PT begins to increase, thus forming a negative sharp piezo-pyroelectric current in the external circuit. As the temperature of the PMN-PT further decreases to room temperature, the negative pyroelectric current gradually vanishes. Figure 4c shows a representative output current signal when the PMN-PT sensor is stimulated by a cooled thermoelectric module, and Figure 4d displays the corresponding current generation mechanism. When the cooled thermoelectric module approaches the PMN-PT sensor, heat is transmitted from the PMN-PT to the cooled thermoelectric module, leading to a negative pyroelectric current. When the cooled thermoelectric module is in contact with the PMN-PT sensor, the force-induced compression and cooling-induced temperature changes exert opposite effects on the spontaneous polarization strength of the PMN-PT. Consequently, piezoelectric current and pyroelectric current with opposite polarities are formed, leading to a small positive piezo-pyroelectric current at the beginning of the contacting stage. As the PMN-PT is further cooled, the pyroelectric effect starts to play a dominant role, leading to a negative current. When the cooled thermoelectric module is removed, a negative piezoelectric current is generated due to enhanced spontaneous polarization. As the temperature of the PMN-PT reduces to room temperature, the current in the circuit diminishes.

Figure 4e,f illustrate the output current from the PMN-PT sensor under different pressures exerted by heated and cooled thermoelectric modules, respectively. Owing to the distinct differences between the response time of piezoelectric and pyroelectric response, pressure and temperature information can be extracted from the piezo-pyroelectric currents by analyzing the peak current *I*_1_ and plateau current *I*_2_, respectively (Figure 4e,f). For convenience, the temperature difference between the thermoelectric module and the PMN-PT sensor is marked as δ*T*. Figure 4g displays the peak current *I*_1_ as a function of pressure under various δ*T*. Obviously, the peak current *I*_1_ linearly increases with pressure under each δ*T*, indicating the capability of the PMN-PT sensor for pressure sensing even if the touched objects have different temperatures. Additionally, owing to the combination of the piezoelectric and pyroelectric effects, the sensitivity for pressure sensing is higher when the touched object has a higher temperature than the PMN-PT sensor. When the temperature difference δ*T* between the thermoelectric module and the PMN-PT sensor is increased from −7.51 °C to 25.8 °C, the sensitivity for pressure sensing is increased from 22.22 nA kPa^−1^ to 48.83 nA kPa^−1^. Figure S14 illustrates the plateau current *I*_2_ as a function of temperature under different pressures, which shows that the plateau current *I*_2_ strongly depends on δ*T* but remains almost constant at different pressures. Because of the ultra-fast piezoelectric response of the PMN-PT, the generation and disappearance of piezoelectric current occur within tens of milliseconds. Consequently, the plateau current *I*_2_ is seldom influenced by pressure and can be an excellent indicator for temperature sensing. As exhibited in Figure 4h, the sensitivity of the PMN-PT sensor for detecting δ*T* is 17.75 nA °C^−1^. Table S1 presents comparisons of the sensitivities of our PMN-PT bimodal sensor with existing ferroelectric pressure and temperature sensors [21,22,25,45,46,47,48,49,50,51,52]. Our PMN-PT sensor possesses the highest temperature sensitivity and relatively high pressure sensitivity among these devices, highlighting its potential for high-performance multifunctional sensing applications.

### 3.5. Self-Powered Pressure–Temperature Sensing for Robotic Stimuli Classification

**Figure 5:**
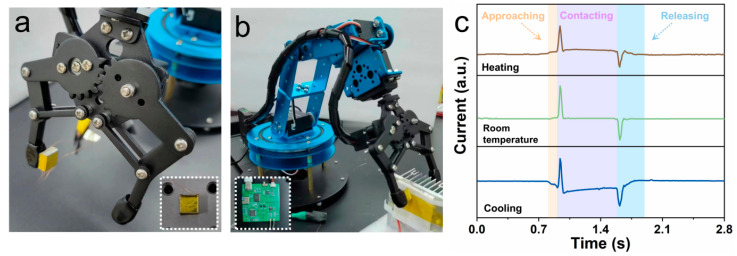

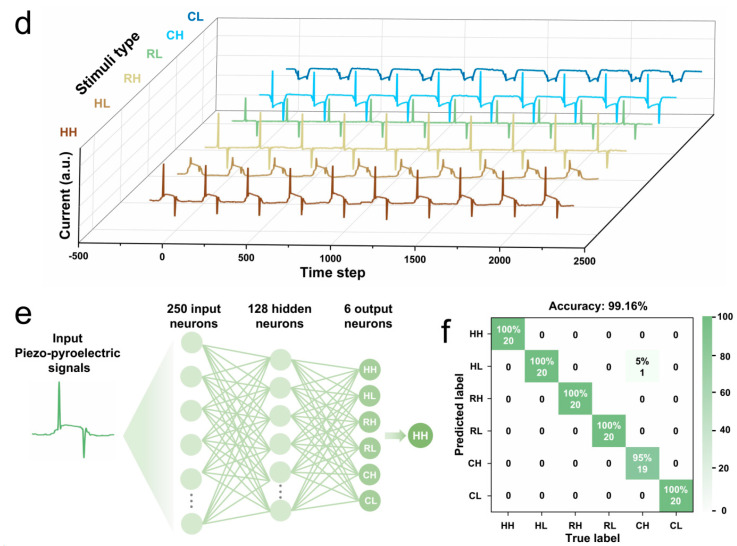
Robotic stimuli classification based on the PMN-PT bimodal sensor. (**a**) Photograph of a robotic hand equipped with the PMN-PT sensor. The insert shows a photograph of the sensor, which is integrated onto an acrylic frame together with a polyimide film. (**b**) Photographic image of the measurement setup. The insert exhibits the customized printed circuit board for signal acquisition and transmission. (**c**) Piezo-pyroelectric current from the PMN-PT sensor as the robotic hand clasps on the thermoelectric module with different temperatures. (**d**) Output current of the sensor as the robotic hand is cyclically stimulated by different types of stimuli. (**e**) Schematic illustration of the fully connected ANN for stimuli classification. (**f**) Confusion map for stimuli classification.

Precise sensing and accurate classification of physical stimuli have been playing a crucial role in the field of robotic perception since they enable robots to achieve accurate object manipulation and recognition. To demonstrate the potential of our bimodal PMN-PT sensor for robotic perception, we integrated it into a robotic hand to simultaneously acquire pressure and temperature information. Furthermore, by utilizing machine learning techniques, we performed stimuli classification based on the acquired pressure and temperature data. Before being attached to a robotic hand, the PMN-PT bimodal sensor is covered with a thin polyimide film using silver paste and integrated into an acrylic frame. This design ensures the electrical insolation of the sensor from touched objects, thus avoiding leakage current. The low thermal conductivity of the acrylic frame can prevent heat transmission from the PMN-PT sensor to the robotic hand, enabling a high-temperature response of the sensor. Figure 5a displays a photograph of the PMN-PT sensor-equipped robotic hand, and the inset shows the integrated PMN-PT sensor. To acquire various stimuli, a thermoelectric module at different temperatures is placed between the fingers of the robotic hand. By controlling the clamp of the robotic fingers, six types of stimuli including “hot and high-pressure” (HH), “hot and low-pressure” (HL), “room-temperature and high-pressure (RH)”, “room-temperature” and “low-pressure” (RL), “cold and high-pressure” (CH), “cold and low-pressure” (CL) can be exerted on the PMN-PT sensor. Photographic images of the measurement setup and the printed circuit board for signal acquisition are shown in Figure 5b. Figure 5c exhibits the typical time-dependent current response of the PMN-PT sensor when the robotic hand touches the thermoelectric module at different temperatures. The three-stage current responses are consistent with the results from a bared Ag/PMN-PT/Ag device (Figure 4a,c). With periodically clamping and unclamping the robotic hand, stable current responses can be obtained (Figure 5d), indicating the high reliability of the whole system. The output current of each type of stimuli is tested 100 times to build reliable datasets. The samples were randomly divided into training and validation datasets with a ratio of 8:2. A fully connected ANN model, known as a multilayer perceptron (MLP) is employed to achieve stimuli classification. The constructed ANN is composed of 250 input neurons (corresponding to the time step number of each current response), a single hidden layer with 128 neurons, and 6 output neurons (corresponding to 6 stimuli types), as illustrated in Figure 5e. The implementation of the ANN model is carried out using the PyTorch framework and trained through backward propagation using the stochastic gradient descent method with a cross-entropy loss function. Figure S15 illustrates the dependence of accuracy (Figure S15a) and loss (Figure S15b) during the training/testing processes on the epoch. The testing accuracy reaches saturation after only 15 epochs, achieving a high recognition accuracy of 99.16%. Consequently, nearly all stimuli can be correctly classified, as exhibited in the confusion matrix in Figure 5f.

## 4. Conclusions

In summary, we demonstrate a self-powered pressure–temperature bimodal sensor based on the large piezo-pyroelectric effect of ferroelectric PMN-PT single-crystal materials. The PMN-PT sensor exhibits rapid and sensitive responses to changes in pressure and temperature. The distinct differences in piezoelectric and pyroelectric responses allow the PMN-PT sensor to simultaneously capture pressure and temperature information from mixed-force thermal stimuli. Moreover, the designed bimodal PMN-PT sensor enables a robotic hand to perceive pressure and temperature, enabling highly precise stimuli classification. The compact device structure and self-powered property of the sensor make it conducive to constructing low-consumption and high-integration electronic networks. This work paves the way for the development of multifunctional self-powered sensors based on PMN-PT materials, pushing the advancement of low-consumption artificial perception forward.

## Data Availability

Data are contained within the article.

## Supplementary Materials

The following supporting information can be downloaded at: https://www.mdpi.com/article/10.3390/s24092773/s1. Figure S1. Ferroelectric properties of the PMN-PT single crystal. Figure S2. Dielectric properties of the PMN-PT single crystal. Figure S3. A photograph showing the piezoelectric constant *d*_33_ of the Ag/PMN-PT/Ag device. Figure S4. Absolute values of pyroelectric current and the corresponding pyroelectric coefficient *P*_C_ of the PMN-PT sensor as its temperature is changed by Δ*T.* Figure S5. Response and recovery time of the PMN-PT bimodal sensor for pressure monitoring. Figure S6. Piezoelectric current of the PMN-PT bimodal sensor with different external resistance. Figure S7. Dependence of the positive piezoelectric current and the corresponding output power of the PMN-PT bimodal sensor on the external resistance. Figure S8. Long-term stability of the PMN-PT sensor for pressure monitoring. Figure S9. Working mechanism of the sensor under cooling conditions. Figure S10. Time-dependent temperature gradient Δ*T* exerted on the PMN-PT sensor (−7.8 °C to 10.00 °C). Figure S11. Response time of the PMN-PT sensor for temperature sensing. Figure S12. Electrical impedance of the bimodal PMN-PT sensor. Figure S13. Long-term stability of the PMN-PT sensor for temperature monitoring. Figure S14. The plateau piezo-pyroelectric current *I*_2_ as a function of temperature under different pressures. Figure S15. Dependence of stimuli classification accuracy and loss on epoch during training and testing. Table S1. Comparisons of the sensitivities of ferroelectric pressure and temperature sensors.
