# Supplementary material for: Self-Powered Pressure–Temperature Bimodal Sensing Based on the Piezo-Pyroelectric Effect for Robotic Perception
Source: Sensors (Basel). 2024 Apr 26;24(9):2773. doi: 10.3390/s24092773 (PMC11086114; doi:10.3390/s24092773)
Supplement: Supplementary file 1 [file sensors-24-02773-s001.zip › sensors-2979283-supplementary.pdf]

sensors

MDPI

Supplementary Materials for

Self-Powered Pressure–Temperature Bimodal Sensing Based on the Piezo-Pyroelectric Effect for Robotic Perception

Xiang Yu [1,2,3], Yun Ji [4,*], Xinyi Shen [1,2,3] and Xiaoyun Le [1,2,3,*]

[1] School of Physics, Beihang University, Beijing 100191, China
[2] Beijing Advanced Innovation Center for Big Data-Based Precision Medicine, School of Medicine and Engineering, Beihang University, Beijing 100191, China
[3] Beijing Key Laboratory of Advanced Nuclear Energy Materials and Physics, Beihang University, Beijing 100191, China
[4] Department of Electrical and Computer Engineering, National University of Singapore, 4 Engineering Drive 3, Singapore 117583, Singapore
* Correspondence: jiyun@nus.edu.sg (Y.J.); xyle@buaa.edu.cn (X.L.)

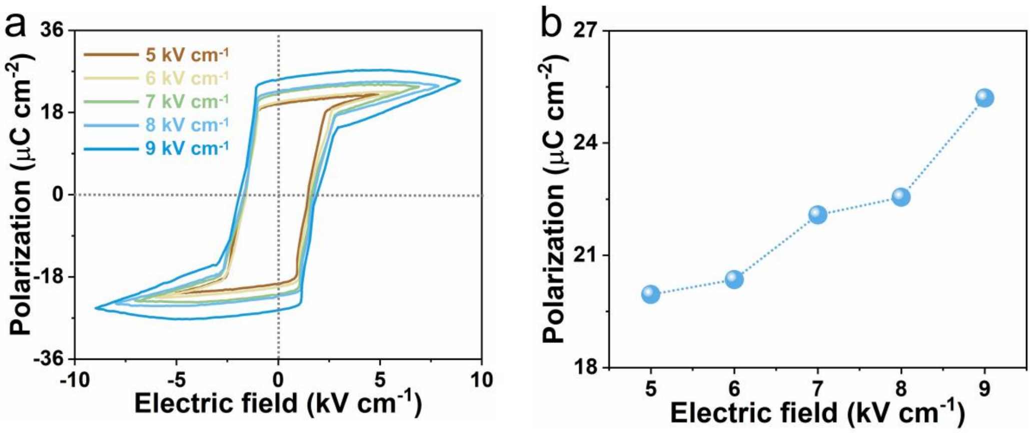

Figure S1. Ferroelectric properties of the PMN-PT single crystal. (**a**) *P-E* hysteresis loops of the PMN-PT. (**b**) Remnant polarization of the PMN-PT as a function of electric field.

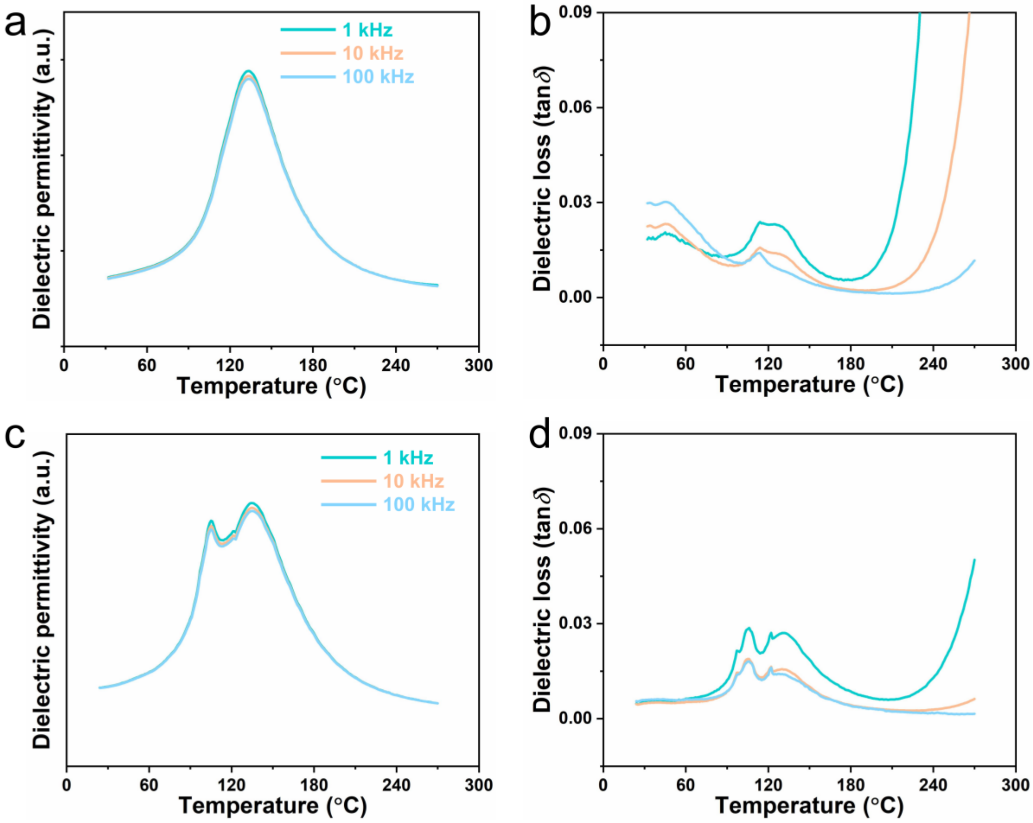

Figure S2. Dielectric properties of the PMN-PT single crystal. (**a**,**b**) Dielectric permittivity (a) and dielectric loss (b) of unpolarized PMN-PT. (**c**,**d**) Dielectric permittivity (c) and dielectric loss (d) of polarized PMN-PT.

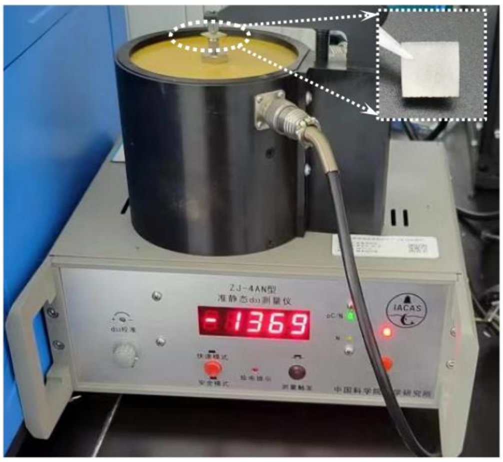

Figure S3. A photograph showing the piezoelectric constant d_{33} of the Ag/PMN-PT/Ag device.

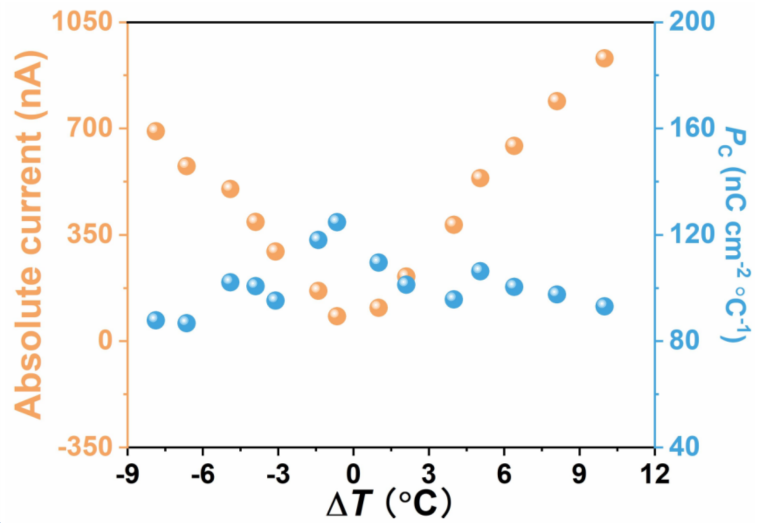

Figure S4. Absolute values of pyroelectric current and the corresponding pyroelectric coefficient P_C of the PMN-PT sensor as its temperature is changed by ΔT.

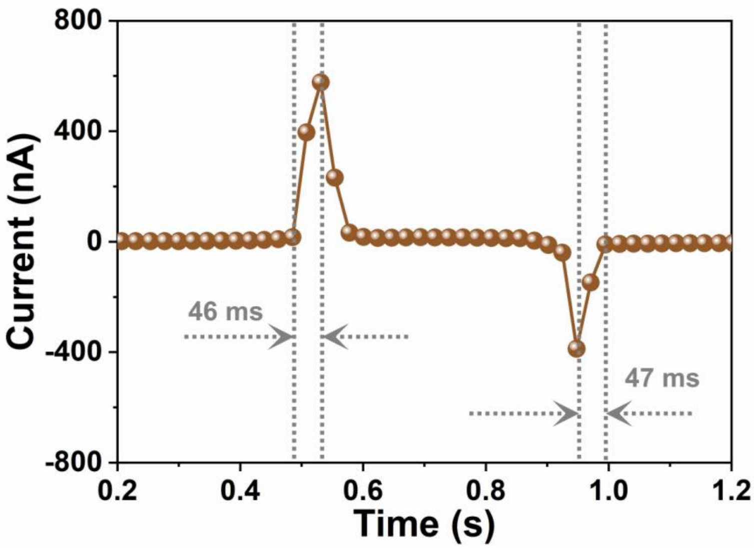

Figure S5. Response and recovery time of the PMN-PT bimodal sensor for pressure monitoring.

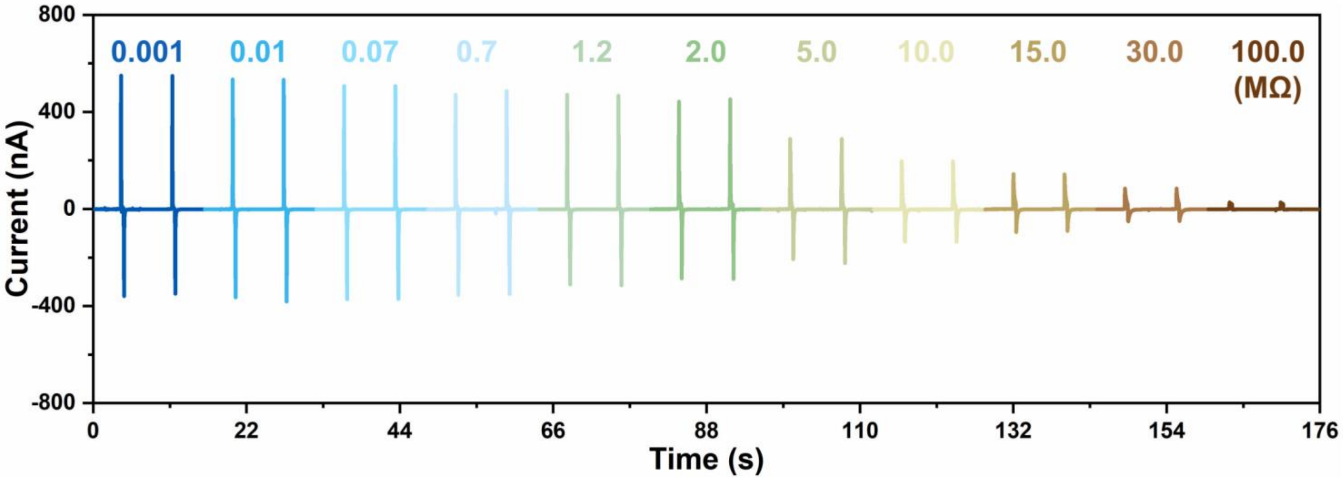

Figure S6. Piezoelectric current of the PMN-PT bimodal sensor with different external resistance.

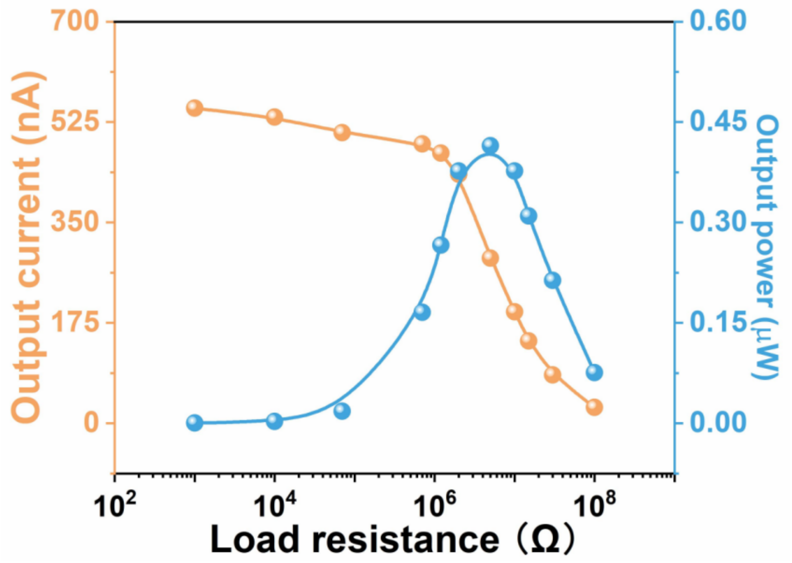

Figure S7. Dependence of the positive piezoelectric current and the corresponding output power of the PMN-PT bimodal sensor on the external resistance.

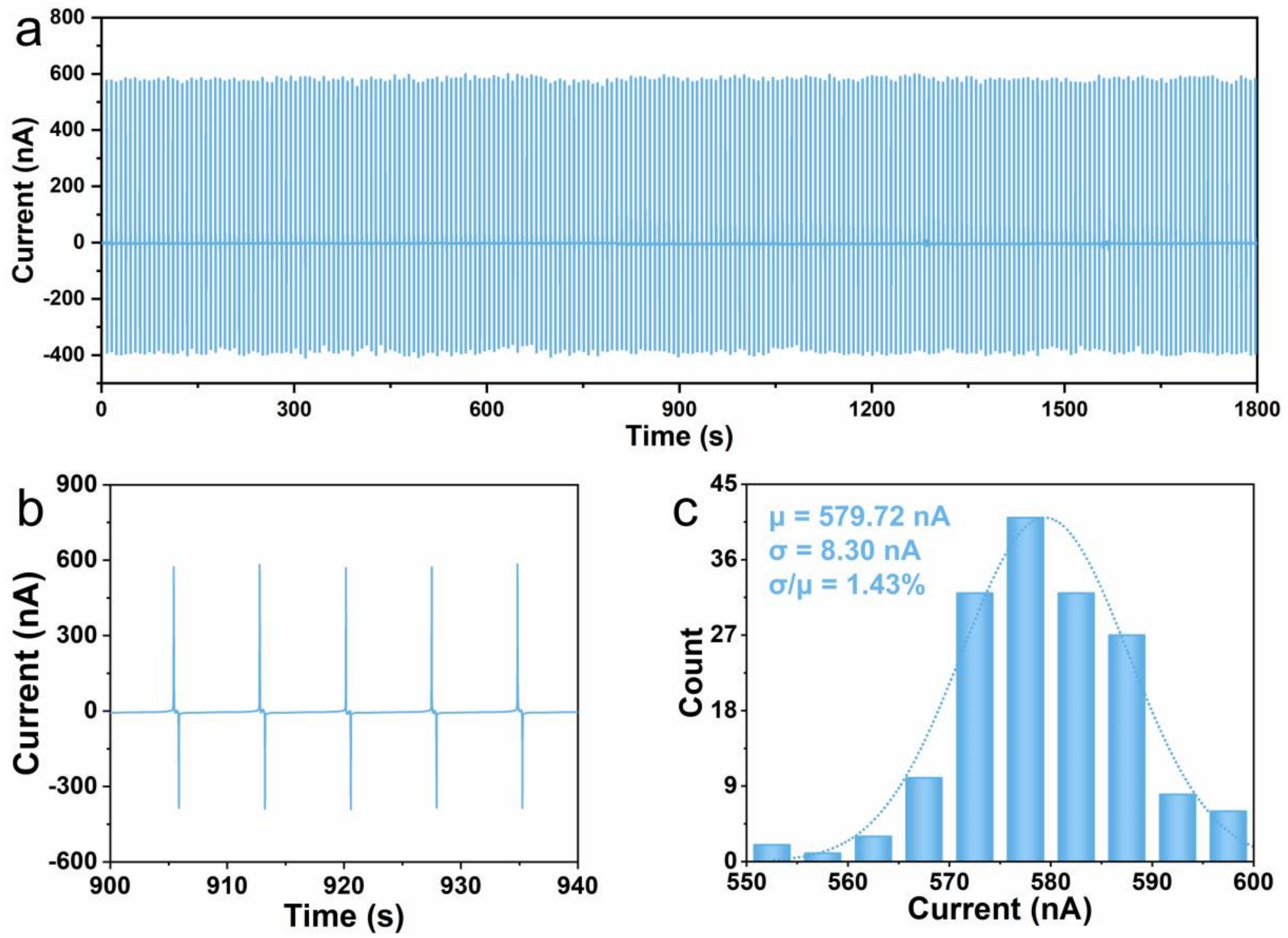

Figure S8. Long-term stability of the PMN-PT sensor for pressure monitoring. (**a**) Stability test of the PMN-PT sensor for pressure sensing under 125.6 kPa for 1800 s. (**b**) An enlarged part of the output current. (**c**) Statistical analysis of the cycle-to-cycle variability of the output current.

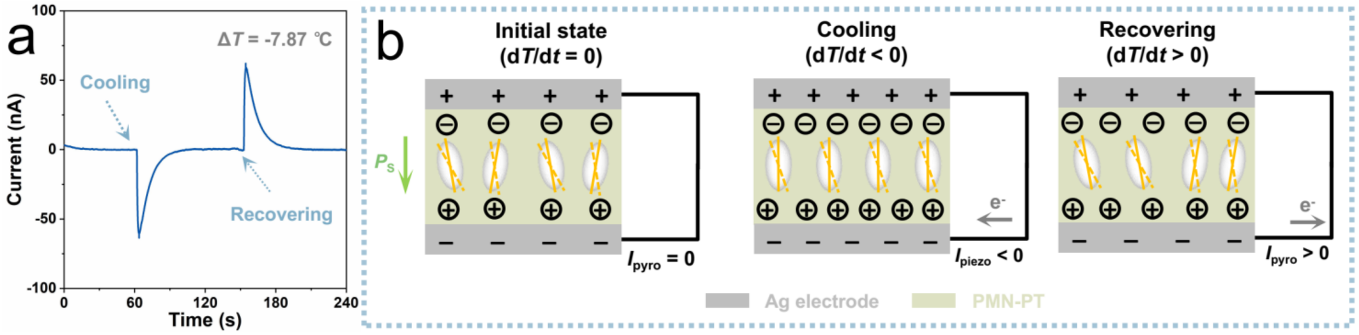

Figure S9. Working mechanism of the sensor under cooling condition. (**a,b**) A typical pyroelectric current signal of the sensor (a) and the corresponding current generation mechanism (b) under cooling stimuli with a temperature gradient ΔT of -7.87 °C.

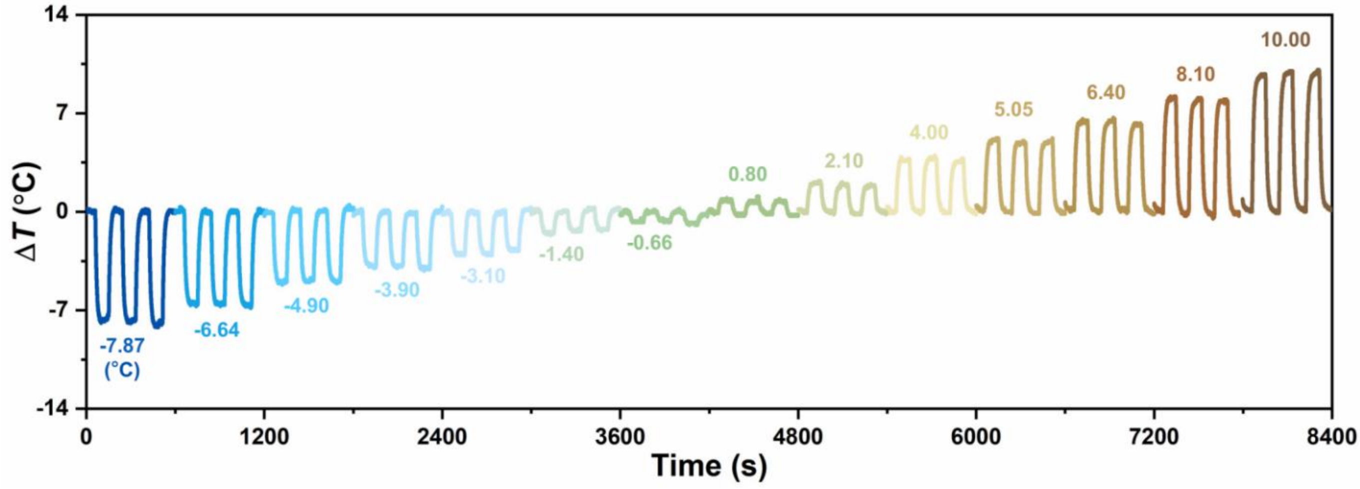

Figure S10. Time-dependent temperature gradient ΔT exerted on the PMN-PT sensor (-7.8 °C to 10.00 °C).

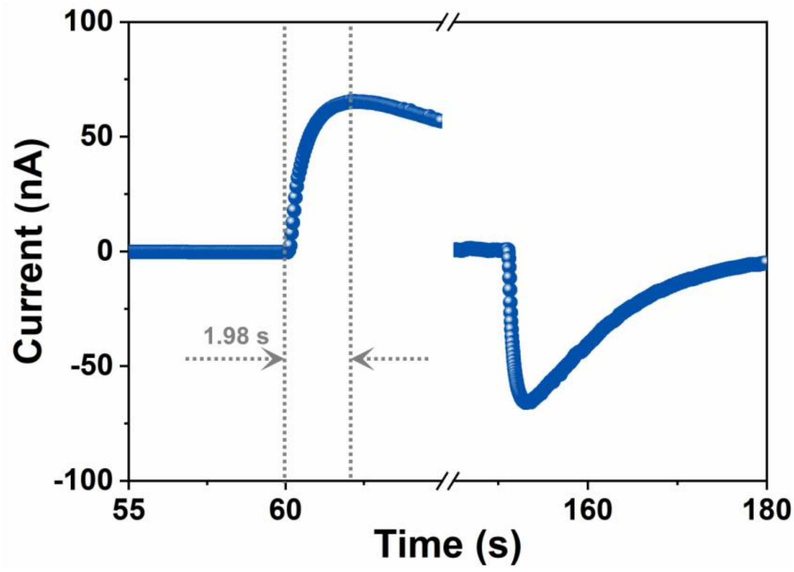

Figure S11. Response time of the PMN-PT sensor for temperature sensing.

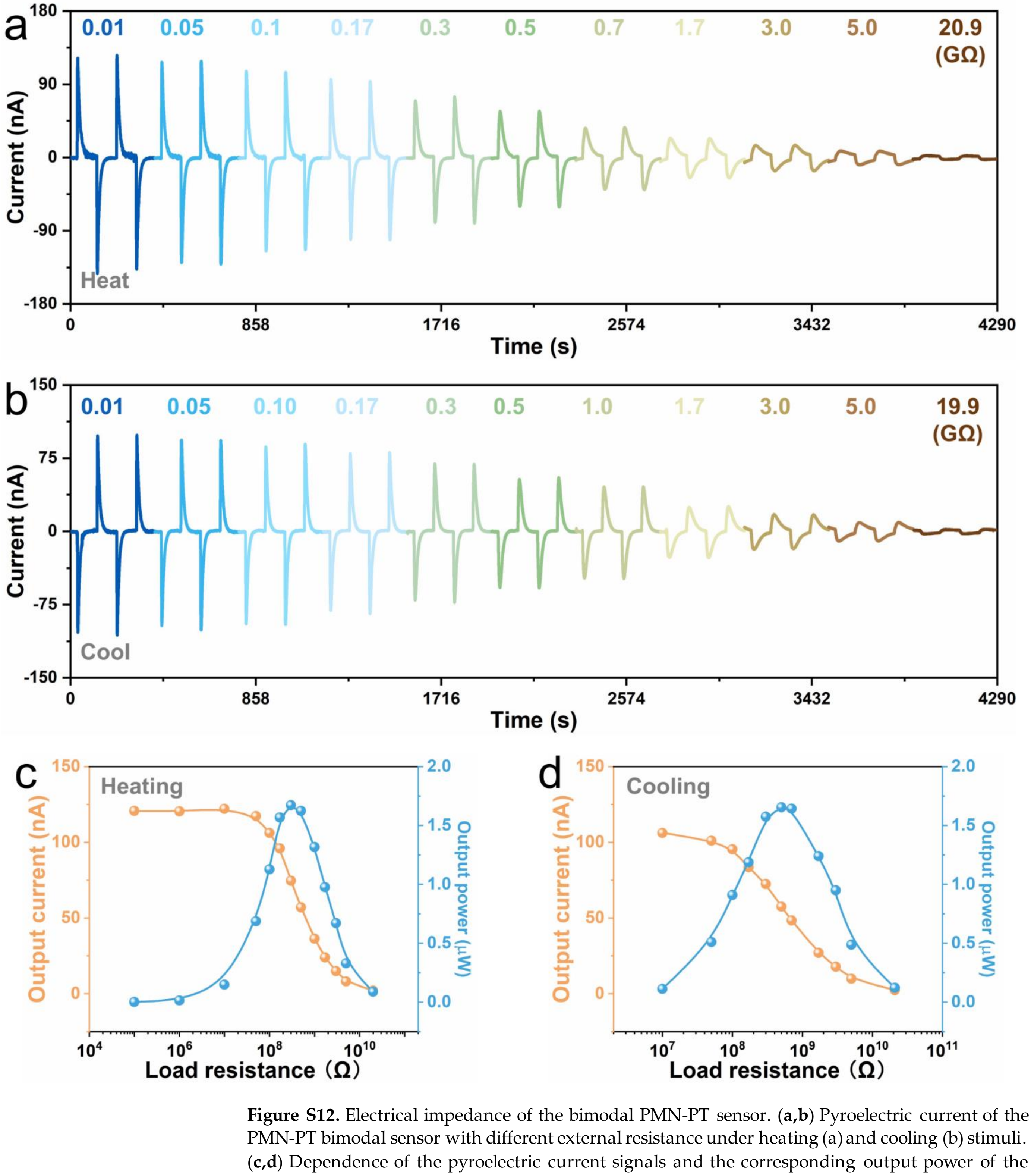

Figure S12. Electrical impedance of the bimodal PMN-PT sensor. (**a**,**b**) Pyroelectric current of the PMN-PT bimodal sensor with different external resistance under heating (a) and cooling (b) stimuli. (**c**,**d**) Dependence of the pyroelectric current signals and the corresponding output power of the PMN-PT bimodal sensor on resistance under heating (c) and cooling (d) stimuli.

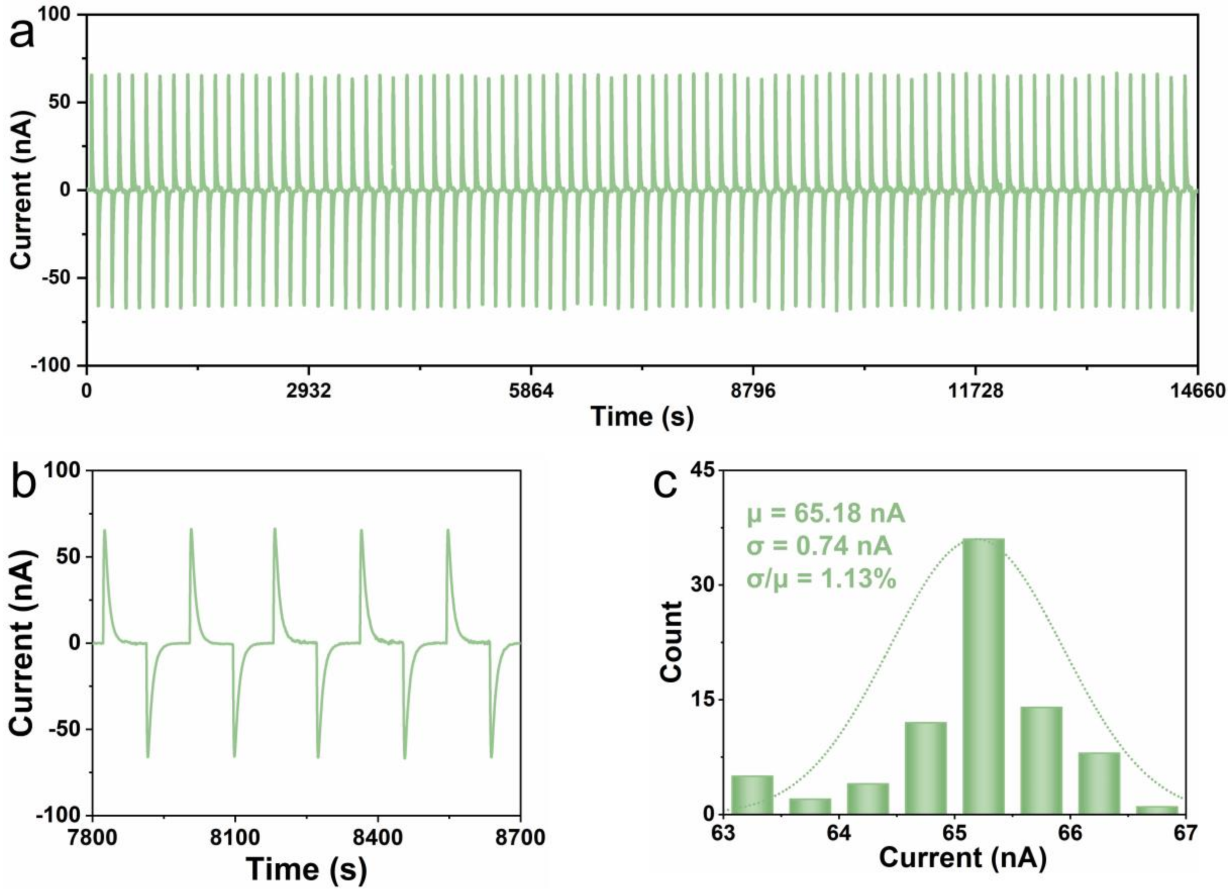

Figure S13. Long-term stability of the PMN-PT sensor for temperature monitoring. (**a**) Stability test of the PMN-PT sensor for temperature sensing under cyclical heating for 14660 s. (**b**) An enlarged portion of the output current. (**c**) Statistical analysis of the cycle-to-cycle variability of the output current.

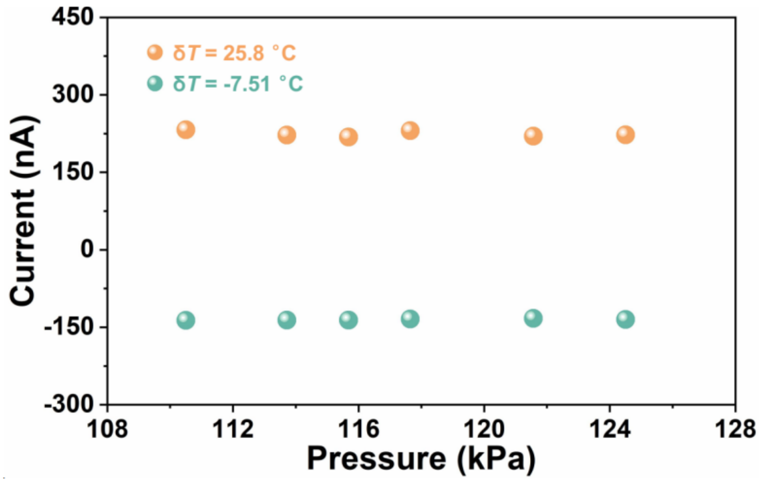

Figure S14. The plateau piezo-pyroelectric current I_2 as a function of temperature under different pressures.

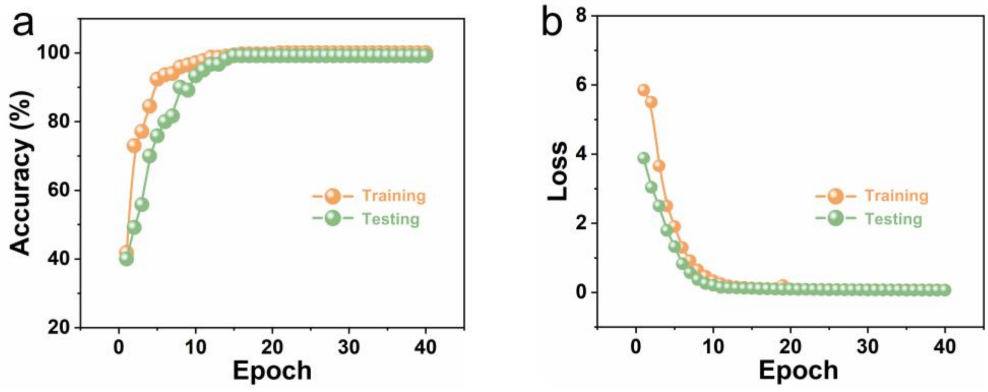

Figure S15. Dependence of stimuli classification accuracy and loss on epoch during training and testing. (**a**) Dependence of accuracy on epoch. (**b**) Dependence of loss on epoch.

Table S1. Comparisons of the sensitivities of ferroelectric pressure and temperature sensors.

Materials	Operation mode	Pressure sensitivity (nA kPa^{-1})	Temperature sensitivity (nA $°C^{-1}$)	Ref.
PMN-PT	Pressure & temperature sensing	28.4	17.75	This work
$BaTiO_3$	Pressure & temperature sensing	–	0.48	[21]
P(VDF-TrFE)	Pressure & temperature sensing	–	0.27	[22]
$BaTiO_3$	Pressure & temperature sensing	1.43	8.85	[25]
P(VDF-TrFE)	Pressure & temperature sensing	40	0.38	[45]
P(VDF-TrFE)	Pressure & temperature sensing	4.6	~0.008	[46]
PVDF	Pressure sensing	11.9	–	[47]
Nylon-11	Pressure sensing	2.82×10^{-3}	–	[48]
$[C(NH_2)_3]ClO_4$	Pressure sensing	20	–	[49]
P(VDF-TrFE)	Pressure sensing	12.4	–	[50]
$BaTiO_3$	Pressure sensing	0.2	–	[51]
$0.94(Bi_{0.5}Na_{0.5})TiO_3$-$0.06Ba(Zr_{0.25}Ti_{0.75})O_3$	Temperature sensing	–	1.4	[52]

References

1. Han, Y.; Song, L.; Du, H.; Wang, G.; Zhang, T.; Ni, L.; Li, Y. Enhancing Structural Response via Macro-Micro Hierarchy for Piezoelectric Nanogenerator and Self-Powered Searable Controller. *Chem. Eng. J.* **2024**, *481*, 148729.
2. Yan, D.; Wang, J.; Xiang, J.; Xing, Y.; Shao, L.-H. A Flexoelectricity-Enabled Ultrahigh Piezoelectric Effect of a Polymeric Composite Foam as a Strain-Gradient Electric Generator. *Sci. Adv.* **2023,** *9*, eadc8845.
3. Xu, Q.; Wang, Z.; Zhong, J.; Yan, M.; Zhao, S.; Gong, J.; Feng, K.; Zhang, J.; Zhou, K.; Xie, J.; et al. Construction of Flexible Piezoceramic Array with Ultrahigh Piezoelectricity via a Hierarchical Design Strategy. *Adv. Funct. Mater.* **2023**, *33*, 2304402.
4. Song, L.; Huang, Z.; Guo, S.; Li, Y.; Wang, Q. Hierarchically Architected Polyvinylidene Fluoride Piezoelectric Foam for Boosted Mechanical Energy Harvesting and Self-Powered Sensor. *ACS Appl. Mater. Interfaces* **2021**, *13*, 37252–37261.
5. Yan, M.; Zhong, J.; Liu, S.; Xiao, Z.; Yuan, X.; Zhai, D.; Zhou, K.; Li, Z.; Zhang, D.; Bowen, C.; et al. Flexible Pillar-Base Structured Piezocomposite with Aligned Porosity for Piezoelectric Energy Harvesting. *Nano Energy* **2021**, *88*, 106278.
6. Jiang, H.; Song, L.; Huang, Z.-X.; Liu, M.; Zhao, Y.; Zhang, S.; Guo, J.; Li, Y.; Wang, Q.; Qu, J.-P. A Novel Concept of Hierarchical Porous Structural Design on Enhancing Output Performance of Piezoelectric Nanogenerator. *Nano Energy* **2022**, *104*, 107921.
7. Li, G.-Y.; Li, J.; Li, Z.-J.; Zhang, Y.-P.; Zhang, X.; Wang, Z.-J.; Han, W.-P.; Sun, B.; Long, Y.-Z.; Zhang, H.-D. Hierarchical PVDF-HFP/ZnO Composite Nanofiber-based Highly Sensitive Piezoelectric Sensor for Wireless Workout Monitoring. *Adv. Compos. Hybrid. Mater.* **2022**, *5*, 766–775.
8. Zeng, W.; Deng, W.; Yang, T.; Wang, S.; Sun, Y.; Zhang, J.; Ren, X.; Jin, L.; Tang, L.; Yang, W. Gradient CNT/PVDF Piezoelectric Composite with Enhanced Force-Electric Coupling for Soccer Training. *Nano Res.* **2023**, *16*, 11312–11319.
9. Hong, Y.; Wang, B.; Long, Z.; Zhang, Z.; Pan, Q.; Liu, S.; Luo, X.; Yang, Z. Hierarchically Interconnected Piezoceramic Textile with a Balanced Performance in Piezoelectricity, Flexibility, Toughness, and Air Permeability. *Adv. Funct. Mater.* **2021**, *31*, 2104737.
10. Li, Y.; Sun, J.; Li, P.; Li, X.; Tan, J.; Zhang, H.; Li, T.; Liang, J.; Zhou, Y.; Hai, Z.; et al. High-Performance Piezoelectric Nanogenerators Based on Hierarchical ZnO@CF/PVDF Composite Film for Self-Powered Meteorological Sensor. *J. Mater. Chem. A* **2023**, *11*, 13708–13719.
11. Kim, D.W.; Kim, H.; Hwang, G.-T.; Cho, S.B.; Jeon, S.H.; Kim, H.W.; Jeong, C.K.; Chun, S.; Pang, C. Conformably Skin-Adherent Piezoelectric Patch with Bioinspired Hierarchically Arrayed Microsuckers Enables Physical Energy Amplification. *ACS Energy Lett.* **2022**, *7*, 1820–1827.
12. Fu, J.Y.; Zhu, W.; Li, N.; Smith, N.B.; Eric Cross, L. Gradient Scaling Phenomenon in Microsize Flexoelectric Piezoelectric Composites. *Appl. Phys. Lett.* **2007**, *91*, 182910.
13. Yang, T.; Deng, W.; Tian, G.; Deng, L.; Zeng, W.; Wu, Y.; Wang, S.; Zhang, J.; Lan, B.; Sun, Y.; et al. Modulating Piezoelectricity and Mechanical Strength via Three-Dimensional Gradient Structure for Piezoelectric Composites. *Mater. Horiz.* **2023**, *10*, 5045–5052.
14. Liu, H.; Lin, X.; Zhang, S.; Huan, Y.; Huang, S.; Cheng, X. Enhanced Performance of Piezoelectric Composite Nanogenerator Based on Gradient Porous PZT Ceramic Structure for Energy Harvesting. *J. Mater. Chem. A* **2020**, *8*, 19631–19640.
15. Malakooti, M.H.; Zhou, Z.; Spears, J.H.; Shankwitz, T.J.; Sodano, H.A. Biomimetic Nanostructured Interfaces for Hierarchical Composites. *Adv. Mater. Interfaces* **2016**, *3*, 1500404.
16. Gottscholl, A.; Diez, M.; Soltamov, V.; Kasper, C.; Krauße, D.; Sperlich, A.; Kianinia, M.; Bradac, C.; Aharonovich, I.; Dyakonov, V. Spin Defects in hBN as Promising Temperature, Pressure and Magnetic Field Quantum Sensors. *Nat. Commun.* **2021**, *12*, 4480.
17. Zhang, F.; Zang, Y.; Huang, D.; Di, C.-a.; Zhu, D. Flexible and Self-Powered Temperature-Pressure Dual-Parameter Sensors Using Microstructure-Frame-Supported Organic Thermoelectric Materials. *Nat. Commun.* **2015**, *6*, 8356.
18. Wang, Y.; Wu, H.; Xu, L.; Zhang, H.; Yang, Y.; Wang, Z.L. Hierarchically Patterned Self-Powered Sensors for Multifunctional Tactile Sensing. *Sci. Adv.* **2020,** *6*, eabb9083.
19. Wen, L.; Nie, M.; Wang, C.; Zhao, Y.-n.; Yin, K.; Sun, L. Multifunctional, Light-Weight Wearable Sensor Based on 3D Porous Polyurethane Sponge Coated with MXene and Carbon Nanotubes Composites. *Adv. Mater. Interfaces* **2022**, *9*, 2101592.
20. Fu, X.; Zhuang, Z.; Zhao, Y.; Liu, B.; Liao, Y.; Yu, Z.; Yang, P.; Liu, K. Stretchable and Self-Powered Temperature-Pressure Dual Sensing Ionic Skins Based on Thermogalvanic Hydrogels. *ACS Appl. Mater. Interfaces* **2022**, *14*, 44792–44798.
21. Ji, Y.; Wang, Y.; Yang, Y. Photovoltaic-Pyroelectric-Piezoelectric Coupled Effect Induced Electricity for Self-Powered Coupled Sensing. *Adv. Electron. Mater.* **2019**, *5*, 1900195.
22. Shin, Y.-E.; Park, Y.-J.; Ghosh, S.K.; Lee, Y.; Park, J.; Ko, H. Ultrasensitive Multimodal Tactile Sensors with Skin-Inspired Microstructures through Localized Ferroelectric Polarization. *Adv. Sci.* **2022**, *9*, 2105423.
23. Park, J.; Kim, M.; Lee, Y.; Lee, H.S.; Ko, H. Fingertip Skin-Inspired Microstructured Ferroelectric Skins Discriminate Static/Dynamic Pressure and Temperature Stimuli. *Sci. Adv.* **2015**, *1*, e1500661.
24. Zhu, P.; Wang, Y.; Wang, Y.; Mao, H.; Zhang, Q.; Deng, Y. Flexible 3D Architectured Piezo/Thermoelectric Bimodal Tactile Sensor Array for E-Skin Application. *Adv. Energy Mater* **2020**, *10*, 2001945.
25. Zhao, K.; Ouyang, B.; Bowen, C.R.; Wang, Z.L.; Yang, Y. One-Structure-Based Multi-effects Coupled Nanogenerators for Flexible and Self-Powered Multi-Functional Coupled Sensor Systems. *Nano Energy* **2020**, *71*, 104632.
26. Ghosh, S.K.; Park, J.; Na, S.; Kim, M.P.; Ko, H. A Fully Biodegradable Ferroelectric Skin Sensor from Edible Porcine Skin Gelatine. *Adv. Sci.* **2021**, *8*, 2005010.
27. Zirkl, M.; Sawatdee, A.; Helbig, U.; Krause, M.; Scheipl, G.; Kraker, E.; Ersman, P.A.; Nilsson, D.; Platt, D.; Bodö, P.; et al. An All-Printed Ferroelectric Active Matrix Sensor Network Based on Only Five Functional Materials Forming a Touchless Control Interface. *Adv. Mater.* **2011**, *23*, 2069–2074.

28. Roy, K.; Ghosh, S.K.; Sultana, A.; Garain, S.; Xie, M.; Bowen, C.R.; Henkel, K.; Schmeißer, D.; Mandal, D. A Self-Powered Wearable Pressure Sensor and Pyroelectric Breathing Sensor Based on GO Interfaced PVDF Nanofibers. *ACS Appl. Nano Mater.* **2019**, *2*, 2013–2025.
29. Ali, T.A.; Groten, J.; Clade, J.; Collin, D.; Schäffner, P.; Zirkl, M.; Coclite, A.-M.; Domann, G.; Stadlober, B. Screen-Printed Ferroelectric P(VDF-TrFE)-co-$PbTiO_3$ and P(VDF-TrFE)-co-$NaBiTi_2O_6$ Nanocomposites for Selective Temperature and Pressure Sensing. *ACS Appl. Mater. Interfaces* **2020**, *12*, 38614–38625.
30. Lee, J.-H.; Lee, K.Y.; Gupta, M.K.; Kim, T.Y.; Lee, D.-Y.; Oh, J.; Ryu, C.; Yoo, W.J.; Kang, C.-Y.; Yoon, S.-J.; et al. Highly Stretchable Piezoelectric-Pyroelectric Hybrid Nanogenerator. *Adv. Mater.* **2014**, *26*, 765–769.
31. Yao, K.; Chen, S.; Lai, S.C.; Yousry, Y.M. Enabling Distributed Intelligence with Ferroelectric Multifunctionalities. *Adv. Sci.* **2022**, *9*, 2103842.
32. Song, K.; Zhao, R.; Wang, Z.L.; Yang, Y. Conjuncted Pyro-Piezoelectric Effect for Self-Powered Simultaneous Temperature and Pressure Sensing. *Adv. Mater.* **2019**, *31*, 1902831.
33. Chen, C.; Wang, Y.; Li, J.; Wu, C.; Yang, G. Piezoelectric, Ferroelectric and Pyroelectric Properties of (100-x)$Pb(Mg_{1/3}Nb_{2/3})O_3$-x$PbTiO_3$ Ceramics. *J. Adv. Dielectr.* **2022**, *12*, 2250002.
34. Kelly, J.; Leonard, M.; Tantigate, C.; Safari, A. Effect of Composition on the Electromechanical Properties of (1-x)$Pb(Mg_{1/3}Nb_{2/3})O_3$-X$PbTiO_3$ Ceramics. *J. Am. Ceram. Soc.* **1997**, *80*, 957–964.
35. Feng, Z.; Li, H.; Luo, H.; Jin, W. High Electric-Field-Induced Strain Beahvior of Single-Crystal $Pb(Mg_{1/3}Nb_{2/3})O_{3-x}PbTiO_3$ Multilayer Piezoelectric Actuators. *J. Electron. Mater.* **2005**, *34*, 1035–1039.
36. Luo, N.; Zhang, S.; Li, Q.; Yan, Q.; He, W.; Zhang, Y.; Shrout, T.R. PMN-PT Based Quaternary Piezoceramics with Enhanced Piezoelectricity and Temperature Stability. *Appl. Phys. Lett.* **2014**, *104*, 182911.
37. Lv, P.; Qian, J.; Yang, C.; Liu, T.; Wang, Y.; Wang, D.; Huang, S.; Cheng, X.; Cheng, Z. Flexible All-Inorganic Sm-Doped PMN-PT Film with Ultrahigh Piezoelectric Coefficient for Mechanical Energy Harvesting, Motion Sensing, and Human-Machine Interaction. *Nano Energy* **2022**, *97*, 107182.
38. Chen, Y.; Zhang, Y.; Yuan, F.; Ding, F.; Schmidt, O.G. A Flexible PMN-PT Ribbon-Based Piezoelectric-Pyroelectric Hybrid Generator for Human-Activity Energy Harvesting and Monitoring. *Adv. Electron. Mater* **2017**, *3*, 1600540.
39. Bowen, C.R.; Taylor, J.; LeBoulbar, E.; Zabek, D.; Chauhan, A.; Vaish, R. Pyroelectric Materials and Devices for Energy Harvesting Applications. *Energ. Environ. Sci* **2014**, *7*, 3836–3856.
40. Zhao, J.; Zhu, R.; Chen, J.; Zhang, M.; Feng, P.; Jiao, J.; Wang, X.a.; Luo, H. Enhanced Temperature Stability of Compensated Pyroelectric Infrared Detector based on Mn:PMN-PT Single Crystals. *Sensor. Actuat. A-Phys.* **2021**, *327*, 112757.
41. Kandilian, R.; Navid, A.; Pilon, L. The Pyroelectric Energy Harvesting Capabilities of PMN-PT near the Morphotropic Phase Boundary. *Smart Mater. Struct.* **2011**, *20*, 055020.
42. Zhao, X.; Dai, J.Y.; Wang, J.; Chan, H.L.W.; Choy, C.L.; Wan, X.M.; Luo, H.S. Relaxor Ferroelectric Characteristics and Temperature-Dependent Domain Structure in a (110)-Cut $(PbMg_{1/3}Nb_{2/3})_{0.75}(PbTiO_3)_{0.25}$ Single Crystal. *Phys. Rev. B* **2005**, *72*, 064114.
43. Wu, N.; Song, X.; Hou, Y.; Zhu, M.; Wang, C.; Yan, H. Relaxor Behavior of (1-x)$Pb(Mg_{1/3}Nb_{2/3})O_3$-x$PbTiO_3$ Ceramics. *Chin. Sci .Bull.* **2009**, *54*, 1267–1274.
44. Ma, N.; Zhang, K.; Yang, Y. Photovoltaic-Pyroelectric Coupled Effect Induced Electricity for Self-Powered Photodetector System. *Adv. Mater.* **2017**, *29*, 1703694.
45. Shin, Y.-E.; Sohn, S.-D.; Han, H.; Park, Y.; Shin, H.-J.; Ko, H. Self-Powered Triboelectric/Pyroelectric Multimodal Sensors with Enhanced Performances and Decoupled Multiple Stimuli. *Nano Energy* **2020,** *72*, 104671.
46. Liu, W.; Lin, D.; Zeng, W.; Wang, Q.; Yang, J.; Peng, Z.; Zhang, Q.; Zhu, G. A Multifunctional Flexible Ferroelectric Transistor Sensor for Electronic Skin. *Adv. Mater. Interfaces* **2021,** *8*, 2101166.
47. Kim, J.; Jang, M.; Jeong, G.; Yu, S.; Park, J.; Lee, Y.; Cho, S.; Yeom, J.; Lee, Y.; Choe, A.; Kim, Y.-R.; Yoon, Y.; Lee, S. S.; An, K.-S.; Ko, H. MXene-Enhanced β-Phase Crystallization in Ferroelectric Porous Composites for Highly-Sensitive Dynamic Force Sensors. *Nano Energy* **2021,** *89*, 106409.
48. Eom, K.; Na, S.; Kim, J.-K.; Ko, H.; Jin, J.; Kang, S. J. Engineering Crystal Phase of Nylon-11 Films for Ferroelectric Device and Piezoelectric Sensor. *Nano Energy* **2021,** *88*, 106244.
49. Li, W.; Li, C.; Zhang, G.; Li, L.; Huang, K.; Gong, X.; Zhang, C.; Zheng, A.; Tang, Y.; Wang, Z.; Tong, Q.; Dong, W.; Jiang, S.; Zhang, S.; Wang, Q. Molecular Ferroelectric-Based Flexible Sensors Exhibiting Supersensitivity and Multimodal Capability for Detection. *Adv. Mater.* **2021,** *33*, 2104107.
50. Eom, K.; Shin, Y.-E.; Kim, J.-K.; Joo, S. H.; Kim, K.; Kwak, S. K.; Ko, H.; Jin, J.; Kang, S. J. Tailored Poly(vinylidene fluoride-co-trifluoroethylene) Crystal Orientation for a Triboelectric Nanogenerator through Epitaxial Growth on a Chitin Nanofiber Film. *Nano Letters* **2020,** *20*, 6651-6659.
51. Zhou, B.; Li, C.; Zhou, Y.; Liu, Z.; Gao, X.; Wang, X.; Jiang, L.; Tian, M.; Zhou, F.-L.; Jerrams, S.; Yu, J. A Flexible Dual-Mode Pressure Sensor with Ultra-High Sensitivity based on BTO@MWCNTs Core-Shell Nanofibers. *Compos. Sci.Technol.* **2022,** *224*, 109478.
52. Zhao, K.; Zhang, H.; Meng, J.; Chung, C.-C.; Gu, B.-N.; Liu, M.-J.; Zhang, D.; Zhong, M.; Liu, M.; Liu, N.; Lin, C.-J.; Meng, C.; Chueh, Y.-L. Design of Self-Powered Sensors with Excellent Thermal and UV-Light Detections by 0.94$(Bi_{0.5}Na_{0.5})TiO_3$-0.06$Ba(Zr_{0.25}Ti_{0.75})O_3$ Nanoparticles. *Adv. Funct. Mater.* **2022,** *32*, 2204234.
